# Management of ureteric endometriosis associated with hydronephrosis: An Australian case series of 13 patients

**DOI:** 10.1186/1756-0500-3-45

**Published:** 2010-02-25

**Authors:** Ian AR Smith, Michael Cooper

**Affiliations:** 1 Department of Urology, Liverpool and Campbelltown Hospitals, NSW, Australia; 2 Faculty of Medicine, University of New South Wales, NSW, Australia; 3 Faculty of Medicine, University of Western Sydney, NSW, Australia; 4 Department of Obstetrics and Gynaecology, University of Sydney, NSW, Australia

## Abstract

**Background:**

Hydronephrosis is a rare but serious manifestation of ureteric endometriosis.

**Findings:**

One hundred and twenty-six women underwent ureterolysis for ureteric endometriosis betweeen and October 1996 and June 2009. Thirteen of the 126 women were identified as having ureteric obstruction at the time of their procedure and were included in the case series. The median age was 39.5 (30 - 63). Chronic pelvic pain was the most common presenting symptom (53.8%). The point of ureteric obstruction was noted to occur most commonly at a small segment of distal left ureter, where it is crossed by the uterine artery (54%). Seven of the 13 women (53.8%) were successfully managed with ureterolysis only. Three of the 13 women (21.3%) underwent ureterolysis and placement of a double J ureteric stent. Three of the 13 (21.3%) required a segmental ureteric resection. There was one incidence of inadvertent thermal ureteric injury which was managed with a ureteric stent. In all cases the hydronephrosis had resolved at six months follow up.

**Conclusions:**

Our findings support the growing body of literature supporting ureterolysis as the optimal treatment for ureteric endometriosis causing moderate to severe ureteric obstruction.

## Introduction

Endometriosis is a benign disease defined by the presence of ectopic endometrial glands and stroma, often associated with pelvic pain and infertility. The incidence of endometriosis in the reproductive age group is estimated at 3-10% [1].

Endometriosis involving the urinary tract includes the presence of endometrial tissue within or around the bladder, ureters, urethra, or kidney. Ureteric endometriosis is usually unilateral, most commonly involving a small segment of distal left ureter [2,3]. It is often associated with retroperitoneal fibrosis and peri-ureteric cicatrization. It has an incidence of < 1% [3-5].

Ureteric obstruction resulting in hydronephrosis is a rare manifestation of ureteric endometriosis. It occurs as a consequence of intrinsic involvement within the ureter of endometriosis, or from extrinsic compression of the ureter by a pelvic endometrioma [6]. In cases of intrinsic ureteric endometriosis, ectopic endometrial tissue is present within the muscularis propria, lamina propria or ureteral lumen [6] In extrinsic cases endometriosis occurs within the ureteral adventitia and adjacent soft tissues only [6]. Extrinsic involvement is approximately 4 times more common than intrinsic disease [6].

It has been shown that pharmacological management with GnRH analogues or aromatase inhibitors alone, in treating ureteric endometriosis, will not prevent the development of hydronephrosis and renal deterioration [7,8]. Despite this, the optimal surgical management of the obstructed ureteric segment has yet to be defined. The current debate, in cases of hydronephrosis associated with ureteric endometriosis, is whether a primary resection with re-implantation or re-anastomosis should be performed, versus a more conservative ureterolysis of the effected ureteric segment.

Given the rarity of the condition there is limited evidence in the literature. There have been several case series published with relatively small numbers and only short to intermediate follow up. Despite this, there is a growing body of evidence favouring ureterolysis as the treatment of choice [3,4,9,10]. We have retrospectively reviewed the records of 126 patients who underwent ureterolysis for ureteric endometriosis by a single gynaecologist. Of these, 13 had macroscopic hydroureter at laparoscopy. This article discusses the surgical management and follow up of these 13 cases, highlighting the favourability of conservative ureterolysis.

## Methods and materials

### Case-series analysis

A retrospective analysis was performed on a database of patients who underwent surgery for endometriosis from a single gynaecologist between 1996 and 2009. A series of 126 cases from the database were identified as having undergone ureterolysis as a part of their procedure. From these 126 cases, 13 were documented as having macroscopic hydroureter at laparoscopy, and were included within the case series for analysis.

### Surgical Procedure

The patient was placed in a lithotomy position with an indwelling catheter in situ. A direct entry technique utilising either a 5 mm or 10 mm reusable trocar was used to access the peritoneal cavity. Three 5 mm ancillary trocars were placed under direct vision. The upper abdomen was inspected and the pelvis assessed. Concomitant procedures, usually related to the presence of endometriosis were performed as required and are listed in table 1. The ureter was approached from a retroperitoneal aspect at the pelvic brim. High power density monopolar electro surgery was utilised to open the retroperitoneal space and access the normal ureter well above the level of any obstruction. Careful dissection was then performed down to the level of the obstruction which usually correlated closely with the site of the uterine artery as it crosses the ureter. Bi-polar desiccation and division of the uterine artery was utilised as required to gain access to the distal ureter. Careful ureterolysis was performed and the adjacent fibrotic cicatrizing endometriotic tissue was removed.

**Table 1:** Intraoperative findings

Intraoperative details	N = 13
**Endometrioma**	8 (61.5%)

**Concomitant endometriosis sites**	

• Ovarian	9 (69.2%)

• Bowel endometriosis	8 (61.5%)

• Uterosacral ligaments	9 (69.2%)

• Completely obliterated cul-de-sac	5 (38.5%)

• Bladder endometriosis	0

**Concomitant surgical procedures**	

• None	3 (23.1%)

• Low rectosigmoid resection and anastomosis	4 (30.8%)

• Partial vaginectomy	1 (7.7%)

• Hysterectomy	5 (38.5%)

• Unilateral salpingoophrectomy	2 (15.4%)

• Bilateral salpingoophrectomy	5 (38.5%)

• Disc resection of anterior rectum	2 (15.4%)

**Side**	

• Left	7 (53.8%)

• Right	5 (38.4%)

• Bilateral	1 (7.7%)

When ureterolysis was not sufficient to free the ureter, the obstructed segment was excised with cold scissors. An open ureteroureteroneostomy was performed in 1 case, whereby the two ureteric ends were spatulated and anastomosed over a ureteric stent using 4/0 interrupted monocryl sutures. In two cases a psoas hitch was created, and an open ureteroneocystostomy was performed through a submucosal tunnel into the bladder and fixed with interrupted sutures of 4/0 vicryl and monocryl. An 8 Fr infant feeding tube was placed in the ureter as a stent. In all cases the ureteric stent was removed after six weeks.

### Indication for JJ stent: 3 cases

In one case there was poor flow of urine from the left ureteric orifice on check cystoscopy at the completion of procedure. The ureter appeared to be clear of endometriosis and there was flow of contrast on retrograde pyelogram therefore it was felt that a ureteric stent would be appropriate management. In another case, extensive ureterolysis was needed to free the ureter from an overlying endometrioma, so a JJ stent was left in situ to reduce the risk of postoperative ureteric fibrosis. In the third case there was an apparent residual ureteric stenosis after all fibrotic bands of tissue had been dissected off the ureter. Since the ureter appeared free of any fibrotic tissue the decision was made to place a JJ stent rather than resecting the ureteric segment. In all cases a JJ stent was inserted for six weeks.

### Pre-operative assessment

Pre-operative imaging of the renal tract was performed in seven of the thirteen patients in the series. Five had a pre-operative renal tract ultrasound and two a CT IVP (computer tomography intravenous pyelogram). Three of the patients had mild hydronephrosis and four demonstrated moderate to severe hydronephrosis. In six cases, only a pelvic ultrasound was performed as a pre-operative work up, therefore no assessment of the renal tract was established. A baseline serum creatinine was taken for all the patients in the series.

### Follow up imaging of the renal tract

All patients were followed up at 3 and 6 months with imaging of the renal tract to assess for residual hydronephrosis. Patients that were managed with ureterolysis only were followed up with a renal tract ultrasound at 3 months. Those that underwent ureterolysis and insertion of a JJ ureteric stent were followed up with a DTPA (Diethylene Triamine Penta-acetic acid) scan at 3 months and a renal tract ultrasound at 6 months, while those who underwent a segmental ureteric resection were followed up with a DTPA scan with frusemide, and a renal tract ultrasound at 6 months.

## Results

### Baseline characteristics

A retrospective case series analysis was performed on 13 women with ureteric endometriosis between 1996 and 2009. The median age was 39.5. The most common presenting symptom was pelvic pain (53.8%), followed by dysmenorrhoae (30.8%), and dyschezia (15.4%). The referral source for the majority of women (70%) was their general practitioner. Three patients in the series had mild hydronephrosis and four had moderate to severe hydronephrosis.

### Operative details

In 12 of the 13 cases, the procedure was attempted laparoscopically. The case that was commenced as an open operation, was done so because of known concurrent rectal endometriosis, requiring an open anterior resection. The aetiology of the ureteric obstruction in nine cases (69.2%) was observed to a fibrotic band of tissue overlying the ureter resulting in stricturing. In two of the cases (15.3%) an endometrioma, and in one case (7.7%) an endometriosis nodule overlying the ureter, had resulted in extrinsic compression. Other intraoperative findings are demonstrated in table 1.

Seven of the thirteen cases were managed with ureterolysis only (53.8%), while three of the cases underwent ureterolysis and insertion of a JJ ureteric stent (23.1%). Only three of the thirteen patients underwent a segmental ureteric resection (21.3%). In the three cases where a segmental ureteric resection was performed, the histopathology demonstrated endometriosis extrinsic to the ureter. The histopathology of all resected tissue confirmed the diagnosis of endometriosis.

### Open conversion

In the series 5 out of the 13 cases were converted to open procedures (42%). These accounted for all the open conversions out of the 126 cases (4%). The reasons for open conversion are outlined below.

#### Case 1

The vast majority of the ureterolysis and pelvic endometrioma dissection was performed laparoscopically. Extensive concomitant bowel disease was encountered that could not be dealt with laparoscopically. An Ileocaecal and low anterior resection were required.

#### Case 2

Extensive pelvic endometriosis was noted involving the rectum and sigmoid colon, both ureters, uterus, and vagina. The patient underwent an open low anterior resection, hysterectomy, partial vaginectomy, and bilateral ureteric segmental resection with the formation of a psoas hitch and ureteroneocystostomy.

#### Case 3

During laparoscopic dissection of an endometrioma off the Iliac vein there was a power surge and temporary loss of vision. When vision was restored bleeding was encountered from a small hole in the internal iliac vein requiring immediate open conversion. This was oversewn with 5/0 proline. This patient also required a left ureteroneocystostomy.

#### Case 4

After access to the peritoneal cavity was achieved it was noted that the pelvis completely filled with large obstructing bilateral endometriomas that were distorting the broad ligaments. This made laparoscopic progress very difficult and the decision was made to convert to an open procedure. This patient underwent a low anterior resection.

#### Case 5

After performing laparoscopically ureterolysis to free the right ureter from the pelvic brim to parametrium further progression was limited by minimal operating space within the pelvis due to a large endometrioma and fixed uterus, and, minimal vision. A distal segment of ureter was resected along with the endometrioma and a ureteroureteroneostomy was performed.

### Follow up imaging

All three patients who underwent a ureteric re-implantation or anastomosis had normal follow up DTPA (Diethylene Triamine Penta-acetic acid) scan and renal tract ultrasound at six months. The patients who were managed with ureterolysis and a JJ stent had a normal renal tract ultrasound or, CT IVP (computer tomography intravenous pyelogram) at six months, while the patients who underwent ureterolysis only all had a normal a renal tract ultrasound at six months. All follow up creatinine measurements were normal.

## Discussion

Laparoscopy is widely accepted as the gold standard of surgical management of ureteric endometriosis due to the enhanced vision [3-5,10-12]. High power density monopolar electrosurgical instruments, combined with excellent vision have meant that laparoscopic dissection around the ureter can occur with minimal complications [3,4,10].

In cases of ureteric obstruction, controversy exists as to whether the effected ureteric segment should be primarily resected, or managed with conservative ureterolysis. Few details have been given as to the actual operative findings, and many cases of obstruction have been managed with ureteric re-implantation [11]. We have retrospectively reviewed the records of 13 cases, and it is our feeling that the majority of patients have an obstruction at the level of the uterine artery, as it crosses the ureter. Endometriosis is a cicatrizing disease and we have been impressed that the disease process often seems to follow the course of adjacent blood vessels such as the uterine artery. The close proximity of the ureter to the uterine artery means that any cicatrizing process adjacent to or surrounding the artery will result in obstruction of the ureter. The fibrotic process forms a constricting band over the ureter and the distal ureter is often free of disease and therefore most of these situations should be resolved with simply excising the affected tissue without recourse to re-implantation.

The current literature relating to hydronephrosis secondary to ureteric endometriosis, consists of mostly case reports and several recent case series. Ghezzi et al (2007), have reported a prospective multi-centre cohort study involving 33 patients with a median follow up of 16 months. In this study only patients with moderate to severe hydronephrosis were included. These authors reported that 85% of the patients in their cohort were successfully managed with ureterolysis as the primary procedure [5]. Mereu, et al 2008, in a single-centre prospective case series with fifty-six patients, all with moderate to severe hydronephrosis, only performed ureterolysis in 62% of cases, while 38% underwent a ureteric resection [4]. Schneider et al (2006) have reported the findings of a prospective case series consisting of 22 women with urinary tract endometriosis. In seven of the 22, endometriosis involved the ureter, including six with distal ureteral endometriosis, and one with endometriosis involving a ureteral stump. Four of the women were suffering mild renal impairment at the time of diagnosis. In this series, six of the seven women underwent segmental ureteric resection with psoas hitching and re-implantation, and excision of the ureteral stump. These authors report no long term complication of relapse at 20 months follow up [13].

In our series ten of the patients underwent ureterolysis alone and three were managed with ureterolysis and a temporary JJ ureteric stent. Therefore 77% of the cohort were successfully treated without resection of the ureteric segment. Of the three patients that were managed with a ureteric resection, two underwent a psoas hitch with a submucosal tunnel of the ureter into the bladder, while one underwent a primary ureteric re-anastomosis. In these cases ureterolysis alone was not sufficient to free the ureter of disease therefore a segmental resection was performed. All of the patients in this series demonstrated a resolution of hydronephrosis at six months follow up.

The seemingly high open conversion rate of 42% reported in this series can be explained by the extensive degree of endometriosis seen in these patients and the need for concomitant procedures. There were two complications in this series of 126 patients (1.5%). These were, one inadvertent ureteric injury that was managed with a ureteric stent, and an injury to the iliac vein which required open conversion. No long term complications occurred.

Histological examination of the three resected ureteric specimens revealed extrinsic endometriosis. This finding is in keeping with the literature, whereby the vast majority of cases have demonstrated extrinsic ureteric endometriosis, and should therefore be suitable for ureterolysis as primary management [3,4,6,14].

When dealing with uncommon disorders it is difficult to evaluate the efficacy of the evidence. The limitations stem from small cohort numbers, due to the rarity of the condition. The majority of evidence in the literature consists of case series, and therefore subject to bias. Moreover, the natural history of endometriosis is largely unknown. If it tends to become inactive then this would favour a ureteric conserving approach.

A major limitation of this study was the short term follow up and no conclusion can be made regarding the risk of recurrent disease. Despite this the data presented in this case series favours ureterolysis as a primary management of ureteric endometriosis, with minimal morbidity.

## Competing interests

The authors declare that they have no competing interests.

## Authors' contributions

In the writing of this paper IS was responsible for the collation of all data, literature search, and the planning and writing of the first paper draft. MC was responsible for the acquisition of the original data and contributed to the intellectual content and writing of the first submitted article. IS was responsible for the writing of the resubmission paper and MC edited the resubmission article. All authors read and approved the final manuscript.

## Authors' information

Dr Ian AR Smith is Associate Conjoint Lecturer in Faculty of Medicine at the University of New South Wales and University of Western Sydney. Dr Michael Cooper is Clinical Associate Professor in the Department of Obstetrics and Gynaecology at the University of Sydney.
